# Metabolic enzymes *aldo-2* and *pdhb-1* as potential epigenetic regulators during *C. elegans* embryogenesis

**DOI:** 10.17912/micropub.biology.001222

**Published:** 2024-06-13

**Authors:** Sonia Ravanelli, Ji Young Cecilia Park, Chantal Wicky, Collin Y. Ewald, Ferdinand von Meyenn

**Affiliations:** 1 Laboratory of Nutrition and Metabolic Epigenetics, Institute for Food, Nutrition and Health, Department of Health Sciences and Technology, ETH Zurich, Switzerland; 2 Laboratory of Extracellular Matrix Regeneration, Institute of Translational Medicine, Department of Health Sciences and Technology, ETH Zurich, Switzerland; 3 Department of Biology, University of Fribourg, Switzerland

## Abstract

The intersection of metabolic processes and epigenetic regulation during embryogenesis is crucial yet not fully understood. Through a candidate RNAi screen in *Caenorhabditis elegans* , we identified metabolic enzymes ALDO-2 and PDHB-1 as potential epigenetic regulators. Mild alteration of the chromatin remodeler LET-418 /Mi2 activity rescues embryonic lethality induced by suppressing *aldo-2* or *pdhb-1 ,* suggesting a critical role for glucose and pyruvate metabolism in chromatin remodeling during embryogenesis. Given the conservation of central metabolic pathways and chromatin modifiers across species, our findings lay the foundation for future mechanistic investigations into the interplay between epigenetics and metabolism during development and upon disease.

**Figure 1. RNAi screening reveals aldo-2 and pdhb-1 metabolic enzymes as potential epigenetic regulators in C. elegans embryonic development:**
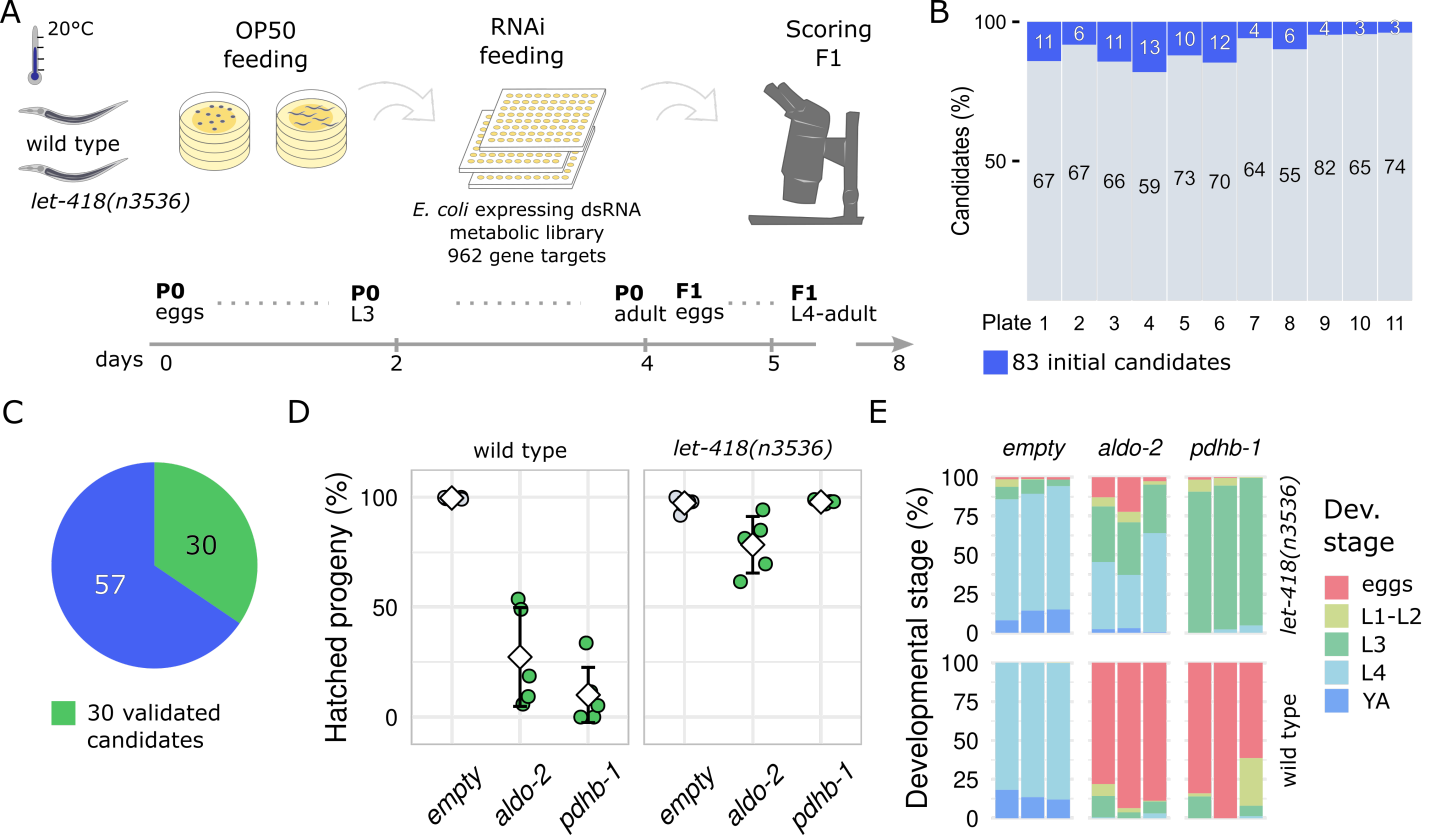
A. Schematic view of the screening setup. B. Identified candidates across the eleven 96-well screening plates seeded with the RNAi metabolic library, excluding control wells. All clones and results of the primary screen are reported in the extended data. C. Validation of the 83 candidates and confirmation by experimental repetition and sequencing of RNAi clones. D. Quantification of embryonic lethality of F2 wild type and *let-418 ( n3536 )* mutant on *aldo-2* and *pdhb-1* RNAi (in green), compared with the empty vector as RNAi control (L4440) (in gray) *.* Means are represented by white squares and standard deviations are shown as error bars. E. Quantification of the developmental rate of F2 wild-type and *let-418 ( n3536 )* animals on empty vector, *aldo-2* , or *pdhb-1* RNAi. Colors indicate the proportion of eggs or larval stages L1-L2, L3, L4, and young adults (YA) present 2 days post-egg-laying.

## Description

Epigenetic events, i.e. heritable changes in gene function that occur without altering the DNA sequence (Berger et al., 2009; Dupont et al., 2009) , can influence DNA accessibility, transcription, replication, and repair. Thus, epigenetic mechanisms provide robust yet flexible cellular responses. These include adaptation to environmental changes and determination of cell identities during embryo development (Cavalli & Heard, 2019; Wilkinson et al., 2023) . The chromatin landscape is shaped by modifications to DNA nucleotides (Iurlaro et al., 2017) and histone amino acids (Y. Zhang et al., 2021), along with ATP-dependent chromatin remodeling enzymes that organize the position and composition of nucleosomes (Eustermann et al., 2024) . Epigenetic mechanisms are known to be intertwined with cellular metabolism – serving as substrates, co-factors, or regulators, metabolites can directly influence the chromatin structure (Z. Dai et al., 2020; X. Li et al., 2018). The chromatin has recently been proposed to act as a metabolic reservoir, supporting the synergy between cellular metabolism and epigenome (Nirello et al., 2022) . Despite vast research, the causal connection between metabolism and epigenetic regulation remains largely unresolved. This study aimed to identify epigenetic regulators among metabolic genes.

The selection of phenotypes exclusively linked with epigenetic defects is challenging due to the multi-level integration of epigenetic processes within complex cellular networks. Cell pluripotency and differentiation states largely depend on their metabolic and epigenetic profiles (Shyh-Chang & Ng, 2017; Wilkinson et al., 2023) . Therefore, developing embryos represent ideal models to elucidate the interaction between metabolic and epigenetic events. Our goal was to identify metabolic genes specifically linked to the epigenetic regulation of embryogenesis and early development. *Caenorhabditis elegans (C. elegans)* is particularly suitable for extensive genetic screening because gene loss-of-function via RNA interference (RNAi) can be efficiently induced by feeding *C. elegans* with bacteria that express double-stranded RNA (dsRNA) homologous to the target gene (Timmons & Fire, 1998) . By specifically suppressing metabolic genes in the temperature-sensitive mutant *let-418 ( n3536 )* , we were able to identify genetic interactors of the chromatin remodeler LET-418 *,* the *C. elegans* homolog of human *CHD3* and *CHD4* (Passannante et al., 2010) *,* respectively known also as *Mi2alpha* and *Mi2beta* .

Mi2 proteins are components of the nucleosome-remodeling and deacetylase (NuRD) complex, which modulates epigenetic processes influencing cell pluripotency and differentiation, as well as proper embryonic development (Alendar & Berns, 2021; Reid et al., 2023) . Loss-of-function mutations in the *C. elegans Mi2 let-418* leads to sterility (von Zelewsky et al., 2000) ; however, animals bearing the temperature-sensitive allele *n3536* generate developmentally arrested progeny at a restrictive temperature of 25 °C, but remain fertile for several generations at a permissive temperature of 20 °C (Käser-Pébernard et al., 2014) . A genome-wide RNAi screen for suppressors of early larval arrest in *let-418 ( n3536 )* predominantly identified chromatin regulators, emphasizing the importance of chromatin remodeling during embryonic and larval development (Erdelyi et al., 2017) . Combining *let-418 ( n3536 )* mutation with a mutation in the histone H3K4 demethylase *spr-5 /LSD1* , which is also fertile for several generations, leads to sterility and germline teratoma at permissive temperature, which enabled the discovery of SPR-5 /LSD1 role in germ cell fate maintenance (Käser-Pébernard et al., 2014) . Inspired by these findings, we employed *let-418 ( n3536 )* mutant at 20 °C as a sensitized background, which appears phenotypically wildtype but bears mild alterations in chromatin structure. To identify metabolic regulators of the epigenome, we fed *let-418 ( n3536 )* and wild-type *C. elegans* with an RNAi library targeting 962 metabolic genes, based on Gene Ontology (GO) term annotations (Melo & Ruvkun, 2012) . Starting RNAi exposure from the L4 stage excluded immediate developmental defects and allowed us to monitor the progeny (F1) development for a few days ( Figure 1A ).

Target genes were scored as candidates when either wild-type or *let-418 ( n3536 )* animals showed defective reproductive fitness. During the initial screening stage, 83 candidates emerged, evenly distributed among the eleven different 96-well screening plates ( Figure 1B ). Of these initial candidates, 40 were validated through three independent repeats, with 30 confirmed to target the correct gene by plasmid sequencing ( Figure 1C ). To identify candidate genes specifically involved in chromatin remodeling during embryogenesis, we focused on the genes whose suppression exhibited different penetrance between wild-type and *let-418 ( n3536 ) C. elegans* . We aimed to identify *let-418* genetic interactors, excluding general fertility defects unrelated to chromatin remodeling. Suppression of two metabolic enzymes, *aldo-2* and *pdhb-1* , showed the most notable differences in reproductive fitness between wild-type and *let-418 ( n3536 )* . While *aldo-2* (fructose bisphosphate aldolase) participates in glycolysis, *pdhb-1* (pyruvate dehydrogenase beta) is involved in the conversion of pyruvate into acetyl-CoA. Surprisingly, *let-418 ( n3536 )* mutants produced more viable progeny than the wild type when treated with RNAi targeting *aldo-2* or *pdhb-1* . For the other 28 validated candidates, no clear and reproducible differences in response to the gene suppression were observed between wild-type and *let-418 ( n3536 )* animals ( Figure 1C ). However, the inherent variability in RNAi suppression and the slightly different numbers of animals per well posed significant limitations in the screening setup for comparing wild-type and *let-418 ( n3536 )* animals. Thus, we focused on two metabolic enzymes *aldo-2* and *pdhb-1* for further investigations.

To exclude maternal effects, we prolonged the RNAi exposure for one generation and observed strong penetrance of embryonic lethality for F2 wild-type animals: approximately 25% and fewer progeny were viable in response to *aldo-2* and *pdhb-1* RNAi, respectively ( Figure 1D ). In contrast, *let-418 ( n3536 )* mutants showed high success in egg hatching both in the control RNAi and in response to *aldo-2* and *pdhb-1* RNAi, with over 75% and approximately 100% of the eggs hatching, respectively. Interestingly, despite the high number of hatched eggs, we observed a clear developmental delay of *let-418 ( n3536 )* mutants treated with *pdhb-1* RNAi, with almost none of the hatched individuals reaching L4 within the observed time frame. In contrast, more *let-418 ( n3536 )* mutants reached L4 and YA stages upon *aldo-2* RNAi, despite a lower number of hatched eggs ( Figure 1D, 1E). While the striking rescue effect of *let-418 ( n3536 )* highlights a novel link between chromatin remodeling and metabolism during embryogenesis, the subsequent developmental defect – more prominent upon *pdhb-1* suppression compared to *aldo-2* – necessitates future studies to elucidate the mechanistic interplay between *let-418* and the two metabolic enzymes. Ideally, these experiments should be confirmed with genetic mutants of *aldo-2* and *pdhb-1* ; however, in line with their essentiality, to our knowledge no viable mutants are currently available. Future experiments should include the generation of new model systems to partially or reversibly suppress *aldo-2* and *pdhb-1* , for example through auxin-inducible degron technology (Zhang et al., 2015) . This method would not only allow to validate the results obtained with RNAi, but also give the opportunity to examine tissue- and stage-specific functions.

Besides the possibility that *let-418 ( n3536 )* rescue effect derives from downstream regulation of gene expression, this study introduces the fascinating possibility that *aldo-2* and *pdhb-1* are essential for their non-canonical functions in chromatin remodeling. During embryogenesis, the glycolytic flux is both temporally and spatially regulated, confirming an important role of cellular metabolism in determining cell identity (Johnson et al., 2003; Oginuma et al., 2017) . We hypothesize that the suppression of *aldo-2* could impair glycolysis, leading to reduced lactate levels, which are notably high in proliferative cells (Vander Heiden et al., 2010) . More than 50% of glucose is converted into lactate by mouse and human blastocysts, even in the presence of oxygen (Ma et al., 2020) . An increasing number of studies have been revealing a significant role of lactate in epigenetic reprogramming, serving also as a substrate for histone lactylation (D. Zhang et al., 2019). Histone lactylation emerged as a mechanism downstream of increased glycolysis that promotes gene regulatory networks orchestrating pluripotency and tissue determination during embryonic development (S.-K. Dai et al., 2022; Galle et al., 2022; L. Li et al., 2020; Merkuri et al., 2024), as well as immunity and muscle regeneration in adults (Desgeorges et al., 2024; D. Zhang et al., 2019) . Further supporting an epigenetic role of aldolases in embryogenesis, muscular aldolase ALDOA has been reported to localize to the nucleus, interact with DNA, and promote cell proliferation (Mamczur et al., 2013; Ronai et al., 1992) .

Regarding *pdhb-1* , we hypothesized that the impaired conversion of pyruvate into acetyl-CoA directly influences histone acetylation. As reported for ALDOA, multiple pyruvate dehydrogenase subunits have been detected within the nucleus, indicating an alternative epigenetic function regulating cell fate decisions through histone acetylation (Kafkia et al., 2022; W. Li et al., 2022; Nagaraj et al., 2017) . Given the potential for defective LET-418 to disrupt the deacetylation capacity of the NuRD complex (Alendar & Berns, 2021; Reid et al., 2023) , the *let-418 ( n3536 )* mutation may mitigate the effects of *pdhb-1* depletion in *C. elegans* by compensating for low acetyl-CoA levels with reduced histone deacetylation. This scenario suggests that embryogenesis could potentially depend on the acetylation of specific histones at specific chromatin loci. Alternatively, echoing the hypothesis that chromatin acts as a metabolic reservoir (Nirello et al., 2022) , a higher availability of acetyl groups on the chromatin deriving from reduced NuRD-dependent deacetylation could provide the necessary amount of acetyl-CoA for embryonic development. It remains unknown if histone lactylation or acetylation are affected upon *aldo-2* or *pdhb-1* suppression and if these changes are heritable. Future studies are required to identify specific chromatin changes that could be affected by impaired glucose and pyruvate metabolism.

In summary, we have identified the metabolic enzymes *aldo-2* and *pdhb-1* as novel genetic interactors of the chromatin remodeler *let-418* . These findings complement previous and recent studies indicating the importance of metabolic enzymes as epigenetic regulators (Boon et al., 2020; Z. Dai et al., 2020; X. Li et al., 2018) . Importantly, core metabolic pathways such as glycolysis and pyruvate metabolism are well conserved from nematodes to humans. Although the components of the NuRD complex have several paralogs in vertebrates compared to lower organisms, the NuRD's function in chromatin remodeling is well conserved from invertebrates to humans (Reid et al., 2023) . Consequently, this study represents a starting point for future research. On one hand, it can help elucidate fundamental mechanisms linking chromatin remodeling and metabolism during embryogenesis. On the other hand, it can uncover specialized molecular pathways that influence tissue-specific functions or pathological conditions, such as metabolic disorders and cancer.

## Methods

***C. elegans* strains and maintenance**

*C. elegans* strains were maintained according to standard procedures (Stiernagle, 2006) , at 15 °C or 20 °C, on nematode growth medium (NGM) agar plates seeded with OP50 *E. coli* as a food source. Experiments were conducted at 20 °C on the following strains: MT14390 – *let-418 ( n3536 )* V and N2 – Bristol strain, which was used as wild-type control.

***C. elegans* synchronization**

*C. elegans* were synchronized by egg-prep (Stiernagle, 2006) . In brief, mixed *C. elegans* populations were washed off the plates with M9, bleached with sodium hypochlorite solution, and, after 3 washing steps with M9, the obtained eggs were seeded on a culture plate. Estimation of the number of eggs was done in triplicates with a 2 μl drop of suspension in M9 to seed appropriate amounts of *C. elegans* in each plate.

**Genetic screening**

The library of RNAi clones ( HT115 ) targeting metabolic genes was generously shared by Gary Ruvkun to Collin Ewald (Melo & Ruvkun, 2012; Venz et al., 2020) . Bacterial clones were copied from glycerol stocks by growing overnight at 37 °C on 86 x 128 mm LB agar plates supplemented with ampicillin and tetracycline to a final concentration of 50 μg/ml and 12 μg/ml, respectively.

The primary screen was conducted in 96-well plates, each scored at least in 2 independent replicates, as described in (Jongsma et al., 2023) . RNAi against selected candidates was repeated in 24-well plates or 3 cm plates; validated candidates were also confirmed to target the correct gene by plasmid sequencing.

RNA interference (RNAi) was performed following the standard feeding method. Each clone was grown in LB supplemented with ampicillin (50 μg/ml) overnight. The following day, cultures were diluted 1:1 with fresh LB supplemented with ampicillin (50 μg/ml), incubated for 2 hours at 37 °C, and concentrated by centrifugation at 4000 rpm for 10 minutes. Pellets were suspended in fresh LB supplemented with ampicillin (50 μg/ml) and IPTG (1 mM). Bacterial suspension was seeded on NGM plates containing 50 μg/ml ampicillin and 1 mM IPTG (8 μl per well in 96-well plates, 80 μl per well in 24-well plates, 300 μl per 3 cm plates).

Focusing on embryogenesis, parental *C. elegans* were allowed to develop without RNAi treatment by distributing synchronized eggs on NGM plates seeded with OP50 and incubated at 20 °C until the animals reached the L3 stage. L3 larvae were washed off the plates, resuspended in fresh M9 to remove OP50 , and redistributed to RNAi plates. Progeny viability and health were monitored under a stereomicroscope for multiple days and wells containing arrested larvae (up to L2), non-hatching eggs, or clear developmental defects were recorded as possible candidates. As a control, bacteria expressing the empty vector pPD129.36 were present in at least one well in each 96-well plate.

**Embryonic lethality and developmental delay quantification**

Synchronized L3 *C. elegans* were treated with RNAi bacteria (control (L4440), *aldo-2* , *pdhb-1* ) as described in the “Genetic Screening” section. 10 adults were picked from the progeny (F1), transferred to fresh RNAi plates to lay eggs, and removed after 6 hours. The following day eggs and hatched larvae (L1/L2) were quantified (F2 generation). After 2 days from egg-laying quantification of the developmental stages reached by each of three F2 generations was conducted, distinguishing among unhatched eggs, L1/L2, L3, L4, and young adults (YA).

## Reagents

*C. elegans* strains are available from CGC: N2 – Bristol wild type, MT14390 – *let-418 ( n3536 )* V

## Extended Data

Description: Complete screening results. Resource Type: Dataset. DOI: 10.22002/ms7c2-j3251
